# Peripheral Regulation of Central Brain-Derived Neurotrophic Factor Expression through the Vagus Nerve

**DOI:** 10.3390/ijms24043543

**Published:** 2023-02-10

**Authors:** Yoko Amagase, Ryuichi Kambayashi, Atsushi Sugiyama, Yoshinori Takei

**Affiliations:** 1 Faculty of Pharmacy, Osaka Medical and Pharmaceutical University, 4-20-1 Nasahara, Takatsuki 569-1094, Japan; 2 Department of Pharmacology, Faculty of Medicine, Toho University, 5-21-16 Omori-nishi, Ota-ku, Tokyo 143-8540, Japan

**Keywords:** adult neurogenesis, BDNF, vagus nerve, peritoneal cells, gut microbiota

## Abstract

The brain-derived neurotrophic factor (BDNF) is an extensively studied neurotrophin es sential for both developing the brain and maintaining adult brain function. In the adult hippocampus, BDNF is critical for maintaining adult neurogenesis. Adult hippocampal neurogenesis is involved not only in memory formation and learning ability, but also mood regulation and stress responses. Accordingly, decreased levels of BDNF, accompanied by low levels of adult neurogenesis, occurs in brains of older adults with impaired cognitive function and in those of patients with major depression disorder. Therefore, elucidating the mechanisms that maintain hippocampal BDNF levels is biologically and clinically important. It has been revealed that signalling from peripheral tissues contribute to the regulation of BDNF expression in the brain across the blood–brain barrier. Moreover, recent studies indicated evidence that neuronal pathways can also be a mechanism by which peripheral tissues signal to the brain for the regulation of BDNF expression. In this review, we give an overview of the current status in the regulation of central BDNF expression by peripheral signalling, with a special interest in the regulation of hippocampal BDNF levels by signals via the vagus nerve. Finally, we discuss the relationship between signalling from peripheral tissues and age-associated control of central BDNF expression.

## 1. Introduction

Adult hippocampal neurogenesis is required for a wide variety of brain functions such as memory formation [1,2], stress responses [3,4] and mood regulation [5]. Accordingly, impairments in adult hippocampal neurogenesis are associated with seizures [6,7], depression [8] and a decline in learning abilities [9]. Thus, maintaining adult hippocampal neurogenesis is critical for preserving normal brain function.

Studies of the mechanisms that maintain adult neurogenesis have focused on the neurogenic niche of the brain, and they revealed the roles of various growth, transcriptional and trophic factors [10,11]. A neurotrophin brain-derived neurotrophic factor (BDNF) in the hippocampus has been reported to be an essential factor for maintaining adult neurogenesis, as mentioned below. Moreover, studies on the effects of exercise indicated the possibility that signalling from peripheral tissues has the potential to regulate hippocampal BDNF expression, at least in late adulthood [12,13]. Indeed, studies using parabiosis, in which blood circulation is shared between old and young mice, have demonstrated that some soluble factors in the blood migrate into the brain to affect adult hippocampal neurogenesis [14]. In neural stem cells (NSCs) of the hippocampus in aged mice, both the DNA methylation status and expression of the corresponding enzyme ten-eleven translocation methylcytosine dioxygenase 2 (TET2) were decreased, which was associated with age-dependent decline in adult hippocampal neurogenesis [14]. The circulation of blood from young mice through the cardiovascular system of aged mice recovered TET2 expression and DNA methylation status in the NSCs of aged mice [14]. This study demonstrated that parabiosis-mediated augmentation of adult hippocampal neurogenesis results from recovered TET2 expression and DNA methylation status in NSCs, at least in part. Molecules in the blood, such as eotaxin-1 (CCL11) [14], growth differentiation factor-11 (GDF11) [15] and the soluble form of vascular cell adhesion molecule-1 (VCAM1) [16] were identified as factors that affect adult neurogenesis across the blood–brain barrier.

Moreover, studies on the gut microbiota demonstrated that peripheral signalling through the vagus nerve can regulate expression of BDNF, thus affecting adult neurogenesis [17]. Recently, we found that intraperitoneal application of the chemokine CX3CL1 promotes BDNF expression and adult neurogenesis in the hippocampus through the vagus nerve [18]. Thus, signalling from peripheral tissues appears to contribute to the regulation of BDNF expression in the brain not only across the blood–brain barrier, but also via neuronal pathways. In this review, we give a brief overview of the regulation of hippocampal BDNF expression by peripheral tissues, with a focus on the vagus nerve-mediated pathway.

## 2. BDNF

BDNF is a neurotrophin regulating the viability and functional integrity of specific neurons. The *Bdnf* gene is expressed beginning in early development and persisting through adult life [19]. BDNF deletion is homozygous-lethal, and *Bdnf^-/-^* mice exhibit gross neurodevelopmental and sensory defects [20]. In mice with heterozygous deletion of the *Bdnf* gene, spatial learning is impaired, as assessed using the Morris water maze test [21], and the proliferation of neural stem cells (NSCs) in the subgranular zone of the hippocampus is significantly decreased compared to with wild-type mice [22]. Moreover, more new-born neurons die in the heterozygous adult mice [22]. A hippocampus-specific knockout of the *Bdnf* gene in mice results not only in decreased adult neurogenesis, but also impaired novel object recognition and spatial learning [23].

Reciprocally, when BDNF expression was chronically stimulated, neurogenesis was significantly increased in the dentate gyrus of the adult hippocampus [24]. A substantial amount of studies indicated that increased BDNF expression augments in vivo proliferation, differentiation, axonal path migration and maturation of NSCs in the subgranular zone of the hippocampus [25,26]. The BDNF is essential for maintaining adult neurogenesis in the hippocampus.

Downregulation of BDNF expression, which is associated with decreased adult hippocampal neurogenesis, occurs in brains of older adults with decreased learning ability [27,28] and those of patients with major depressive disorders [29]. Moreover, decreased levels of BDNF expression in older adults could result in impaired memory, neurodegeneration and other cognitive impairments typical of Alzheimer’s disease [30]. Therefore, it is not only biologically but also clinically important to determine how BDNF expression levels are regulated. However, the mechanisms regulating expression levels of BDNF under physiological and pathological conditions are not fully understood.

## 3. Vagus Nerve and Regulation of Constitutive BDNF Expression in the Hippocampus

The vagus nerve, which is the tenth cranial nerve, transmits information to and from the viscera and brain [31]. It is a paired nerve consisting of sensory (afferent) and motor (efferent) neurons and is involved in maintaining homeostasis as part of the parasympathetic branch of the autonomic nervous system [31]. Involvement of the vagus nerve in the regulation of hunger, satiety, stress responses and inflammation has been previously demonstrated [32,33,34].

Recently, accumulating evidence has demonstrated that the vagus nerve has a pivotal role in the regulation of the BDNF expression in the brain. Gut hormones such as cholecystokinin, GLP-1 and ghrelin bind to their specific receptors on the surface of afferent vagal fibres, regulating BDNF expression in the hypothalamus and contributing to appetite control [32,35]. Furthermore, O’Leary et al. demonstrated that vagotomy, in which gut-related vagal communication was ablated, decreased constitutive levels of BDNF in the hippocampus and diminished adult hippocampal neurogenesis [36]. These findings demonstrated that peripheral signalling through the vagus nerve contributes to the regulation of constitutive levels of BDNF in the hippocampus. The mechanisms inducing vagal tone to regulate basal levels of hippocampal BDNF expression are unknown.

**Figure 1:**
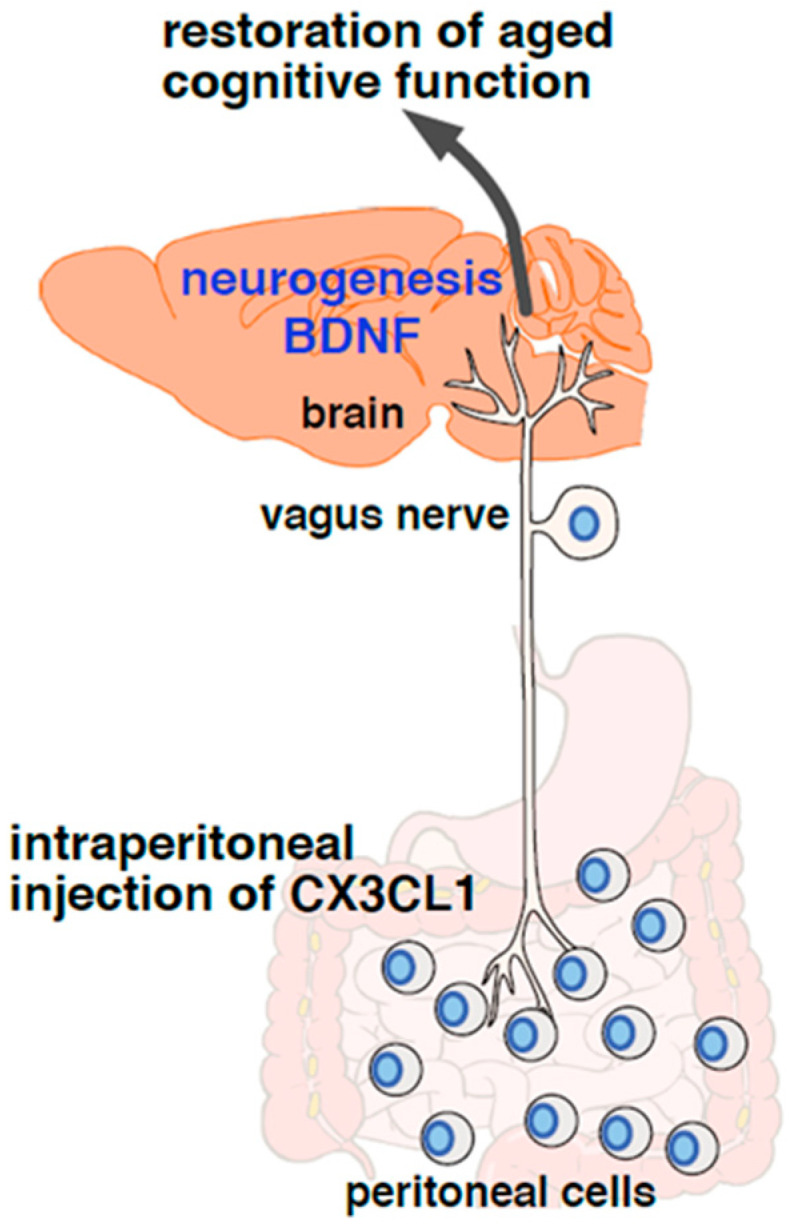
Crosstalk between peritoneal cells and the brain via the afferent vagus nerve. Intraperitoneal administration of the chemokine CX3CL1 partially restores age-associated phenotypic alterations in peritoneal cells, upregulates hippocampal BDNF expression and improves cognition in aged mice. Intraperitoneal transplantation of peritoneal cells prepared from aged mouse donors injected with CX3CL1 into recipient aged mice also improves cognition in recipient aged mice. CX3CL1 injected into the peritoneal cavity thus improves cognition through peritoneal cells. Furthermore, vagotomy abolishes augmentation of BDNF expression induced via intraperitoneal CX3CL1 injection, demonstrating that the vagus nerve is a signalling pathway that connects the peritoneal cells with the function of the hippocampus.

More recently, we found that peritoneal cells transduced signals to the brain through the vagus nerve, increasing hippocampal BDNF expression (Figure 1) [18]. Administration of the chemokine CX3CL1 into the peritoneal cavity of aged mice augmented hippocampal BDNF expression [18]. The CX3CL1-induced BDNF expression was abolished via vagotomy, indicating that the vagus nerve is involved in the signalling pathway from administered CX3CL1 to the hippocampus. As expected from the augmented expression of BDNF in the hippocampus, intraperitoneal administration of CX3CL1 increased the number of hippocampal Type-2 NSCs and improved novel object recognition memory that is impaired by advancing age [18]. Since Type-2 NSCs are thought to be an intermediate stage between radial glial cell-like NSCs and mature neurons [10,37], these results suggested an increase in adult hippocampal neurogenesis. Moreover, CX3CL1 improved age-associated phenotypic changes of peritoneal cells in aged mice, expression of the senescence marker p16*^INK4a^* and phagocytic activity [18]. When peritoneal cells prepared from CX3CL1-treated aged donor mice were transplanted into the peritoneal cavity of recipient aged mice, age-related impairment of novel object recognition memory was improved. This suggests that peritoneal cells are involved in the signalling pathway of CX3CL1-induced BDNF expression in the hippocampus of aged mice.

Vagal afferents primarily project to the tractus solitarius nucleus in the brainstem [38]. While neuronal projection from the tractus solitarius nucleus to the hypothalamus has been well studied [38], projection to the hippocampus is not fully understood. In rats, chronic vagus nerve stimulation via an electric device indicated that signalling from the vagus nerve activated adrenergic neurons in the locus coeruleus and serotonergic neurons in the dorsal raphe nucleus [39,40]. Serotonergic neurons in the dorsal raphe nucleus were required for the augmentation of hippocampal BDNF with chronic vagus nerve stimulation [41].

These findings indicated that signalling from peripheral tissues contributes to the regulation of basal levels of hippocampal BDNF expression through the vagus nerve. Next, we outline findings from studies on exercise and gut microbiota to discuss current knowledge regarding the relationships between signalling from peripheral tissues and hippocampal BDNF expression.

## 4. Exercise Upregulates Hippocampal BDNF Expression

Exercise has beneficial effects on cognition [42,43], which is most prominently observed in the older adults [44]. Moreover, exercise ameliorates symptoms of neurological disorders, such as depression, epilepsy, stroke, Alzheimer’s disease and Parkinson’s disease [45,46,47,48,49]. The effects of exercise on the hippocampus include increases not only in the size of and blood flow to the hippocampus, but also synapse plasticity and adult neurogenesis and induction of morphological changes in dendrites and dendritic spines [42,43,50].

In animal models, moderate exercise induces BDNF expression in various regions of the brain, most robustly in the hippocampus [42]. In mice, voluntary exercise with wheel running increased BDNF levels in the dentate gyrus after only a few days of exercise [51]. These levels were maintained throughout several weeks of exercise [48]. Blocking BDNF signalling inhibits the exercise-induced improvement of acquisition and retention in a spatial learning task [52,53]. While controversial results have been reported with young animals [54], BDNF signalling appears to be associated with the beneficial effects of exercise. The underlying mechanism by which exercise induces hippocampal BDNF expression remains to be determined.

Irisin is a myokine secreted from skeletal muscle during exercise. It consists of 112 amino acids and is cleaved from fibronectin type III domain containing protein 5 (FNDC5) [55]. Exercise increases *Fndc5* gene expression in the skeletal muscle, increasing in circulating irisin [55,56]. Overexpression of *Fndc5* in the liver induces elevation of the blood level of irisin [57], which is associated with increased hippocampal expression of *Bdnf* and other neuroprotective genes [57]. These findings suggest that irisin can be a molecule connecting exercise with hippocampal BDNF expression.

However, exercise also increases FNDC5 expression in various regions of the brain, including the hippocampus [57,58,59]. Forced expression of FNDC5 in primary cortical neurons increases BDNF expression, and reciprocally, knockdown of FNDC5 reduces BDNF. This appears to suggest that FNDC5/irisin expressed in neurons induces BDNF expression in an autocrine and/or paracrine manner. Furthermore, a meta-analysis of twelve studies in eight manuscripts has concluded that chronic resistance exercise training induces a moderate decrease in circulating irisin, while endurance exercise training failed to show a significant difference [60]. It is currently unknown whether irisin secreted from the skeletal muscle after exercise can contribute to the regulation of BDNF expression in the hippocampus.

Cathepsin B is also a myokine of which secretion is upregulated after exercise [61]. Secreted cathepsin B into the circulation can cross the blood–brain barrier [61]; moreover, it runs increased hippocampal expression of cathepsin B and BDNF [61]. Contribution of the vagus nerve to the cathepsin B-induced hippocampal BDNF expression is unknown.

Interestingly, exercise also increases the expression level of CX3CL1 [62] of which intraperitoneal administration increases hippocampal BDNF expression [18]. A secretome analysis of human muscle biopsies demonstrated that 938 gene expressions were altered in the muscle after acute exercise. In those genes, 29 genes encoded putative secreted proteins. CX3CL1 is included in the 29 genes and its increase in plasma levels after exercise was confirmed with an ELISA assay [62]. Therefore, CX3CL1 could also be a candidate for factors connecting exercise with hippocampal BDNF expression.

While there are many candidates for molecules connecting exercise and brain function [63,64], the mechanism is not fully understood. Contribution of the vagus nerve to the exercise-induced BDNF expression in the hippocampus has not been reported.

## 5. Gut Microbiota and BDNF Expression in the Brain

Germ-free mice, which are mice grown up without any exposure to micro-organisms, exhibit reduced BDNF levels in the cortex and hippocampus, and showed increased anxiety-like behaviour (Table 1) [65]. Consistently, depleting gut bacteria in mice via treatment with antibiotics beginning at weaning impairs memory retention and causes a reduction of BDNF in the brain [66]. Probiotic treatment with *Bifidobacterium breve* 6330 from postnatal day 28 to day 70 elicited a two-fold increase in the expression of hippocampal BDNF in normal rats [67]. Oral administration of *Lactobacillus johnsonii* CJLJ103 increased BDNF expression in the hippocampus [68]. When *Lactobacillus plantarum* IS-10506 was administered to rats via a gastric tube daily for 7 days, BDNF levels in the hippocampus increased from 15 µg/mL to approximately 23 µg/mL [69]. Some prebiotics, such as fructo-oligosaccharides and galacto-oligosaccharides, have regulatory effects on BDNF, neurotransmitters and synaptic proteins [70,71]. These reports indicate that the gut microbiota upregulates BDNF levels in the hippocampus.

Contrary to the gut microbiota-dependent elevation of the BDNF levels in the hippocampus, Neufeld et al. demonstrated that germ-free Swiss Webster female mice showed increased BDNF expression in the dentate granule layer of the hippocampus, which was accompanied by anxiolytic behaviour in the elevated plus maze [72]. Oral administration of nonabsorbable antibiotics, such as neomycin, bacitracin and pimaricin, to BALB/c mice altered the composition of the gut microbiota, including increased Lactobacilli, Firmicutes and Actinobacteria and decreased Proteobacteria and Bacteroidetes populations [73]. Coincidentally, the antibiotic treatment increased the BDNF expression in the hippocampus but decreased it in the amygdala. Moreover, the colonisation of germ-free NIH Swiss mice with microbiota from specific pathogen-free grade BALB/c mice exhibited decreased hippocampal BDNF levels and reduced exploratory behaviour [73]. Reciprocally, the colonisation of germ-free BALB/c mice with microbiota from specific pathogen-free NIH Swiss mice increased the exploratory behaviour and hippocampal levels of BDNF [73]. These reports potentially suggest that the composition of the gut microbiota is an important factor for regulation of BDNF levels in the hippocampus.

While these reports demonstrated that probiotics affect BDNF levels in the hippocampus, a meta-analysis of 11 trials (n = 648 participants) indicated that more large-scale, high-quality, randomised controlled trials are needed to make reliable conclusions in their relationship [77].

## 6. Effects of the Gut Microbiota on Major Depression Disorder and Stress Responses

Blood BDNF levels are thought to correlate with BDNF levels in the brain [78]. Decreased BDNF levels in blood are observed in major depressive disorder (MDD) patients, and increased BDNF levels correlate with improved depressive symptoms [79,80]. Besides, adult hippocampal neurogenesis is also implicated in anxiety- and depression-related behaviour. Promoting adult hippocampal neurogenesis through inactivation of the proapoptotic Bax gene in neural progenitor cells decreased anxiety- and depression-related behaviours induced via chronic treatment with corticosterone [81]. Reciprocally, ablation of adult hippocampal neurogenesis with irradiation blocked the antidepressant effects of fluoxetine antidepressant [82]. These reports demonstrate close relationships among BDNF levels, adult hippocampal neurogenesis and anxiety- and depression-associated behaviour.

An animal study demonstrated that antidepressant serotonin-specific reuptake inhibitor increased BDNF expression and adult neurogenesis in the hippocampus, which was accompanied by decreased depressive behaviour [83]. In humans, antidepressants recovered decreased levels of blood BDNF in MDD patients and improved related symptoms [84,85]; BDNF itself has antidepressant effects [86]. Animal studies using a chronic stress model identified that the administration of *Clostridium butyricum* or *Faecalibacterium prausnitzii* attenuated depressive behaviour, which was accompanied by increased BDNF levels [87,88]. In chronic stress-induced depressive mice, *Bifidobacterium longum* subsp. infantis E41 increased BDNF levels and ameliorated depressive behaviour [89]. Several other strains of lactic acid bacteria and bifidobacteria also improve depressive symptoms [90,91]. A randomised, double-blind, placebo-controlled, multicentre clinical trial study with 63 healthy participants over 65 years old, demonstrated that an administration of probiotics containing *Bifidobacterium bifidum* BGN4 and *Bifidobacterium longum* BORI for 12 weeks increased serum BDNF levels and improved mental flexibility and stress scores [92]. Thus, the augmentation of BDNF expression via gut bacteria is effective for improving anxiety- and depression-associated behaviour.

## 7. Mechanisms of Gut Microbiota-Induced Regulation of Brain BDNF Expression

### 7.1. Signalling Pathways to the Brain

Despite the large body of evidence supporting the influence of gut microbiota on BDNF expression in the hippocampus, the precise mechanisms of crosstalk between the gut microbiota and the brain remain incompletely understood. Several mechanisms have been proposed as signalling pathways by which micro-organisms in the peripheral tissues influence processes in the brain. These pathways include signalling pathways via the vagus nerve [93], the immune system [94], the hypothalamic–pituitary-–adrenal (HPA) axis [95,96], short-chain fatty acids (SCFAs) [97,98,99], tryptophan metabolism [100] and bacteria-produced neurotransmitters [97,101,102]. Since this review focuses on the vagus nerve-mediated signalling, we mentioned SCFAs that have been reported to activate the vagus nerve through direct interaction [103,104] and the vagus nerve-mediated pathway.

SCFAs are carboxylic acids with aliphatic tails consisting of 1–6 carbon atoms [105,106,107]. SCFAs are speculated to play a pivotal role in gut microbiota–brain crosstalk. Acetate, propionate and butyrate comprise over 95% of SCFAs produced in the gut [108]. Both acetate and propionate can be digested into butyrate by butyrate-producing bacteria such as *Faecalibacterium prausnitzii* and *Eubacterium rectale* [109,110]. Following production, SCFAs are absorbed by colonocytes, primarily via H^+^-dependent or sodium-dependent monocarboxylate transporters [111]. SCFAs that are not catalysed in colonocytes are transported into the blood and digested in hepatocytes, except for acetate [112]. Therefore, only a small portion of SCFAs produced in the colon reach the other tissues [113]. Nonetheless, uptake of SCFAs into the brain was demonstrated in rats with injection of ^14^C-SCFAs into the carotid artery [114,115]. SCFAs are detectable in human cerebrospinal fluid at an average concentration of 17.0 pmol/mg for butyrate and 18.8 pmol/mg for propionate [116]. Furthermore, the levels of butyrate in mouse brains supplemented with live *Clostridium butyricum* reached a range from 400 to 700 pmol/mg, which was approximately ten times higher than that in the peripheral blood [117,118]. Several other strains of lactic acid bacteria and bifidobacteria also increase butyrate, accompanied by an improvement of depressive symptoms [90,91].

The precise mechanisms for the effects of SCFAs on the brain remain largely unknown. Several studies, however, demonstrated that sodium butyrate, a histone deacetylase inhibitor, has antidepressant-like effects [119,120,121,122]. Wei et al. used a rat depression model to examine the effects of sodium butyrate on DNA methylation in the prefrontal cortex [122,123]. The depressed rats exhibited decreased expression of ten-eleven translocation methylcytosine dioxygenase 1 (TET1), which catalyses the conversion of DNA methylation to hydroxymethylation. The administration of sodium butyrate decreased depressive-like behaviour on the forced swim test and increased TET1 levels, which was negatively correlated with *Bdnf* methylation, subsequently resulting in BDNF upregulation. These findings indicate that the antidepressant effects of sodium butyrate could be mediated via the upregulation of BDNF expression. Butyrate produced by gut microbiota could regulate central BDNF expression through the alteration of DNA methylation.

In addition to the possibility of migration into the brain, SCFAs are reported to activate vagal afferent fibres [103,104]. A study with primary culture of neurons from nodose ganglia demonstrated that butyrate directly interacts with neurons and activates intracellular Ca^2+^ signalling [104]. Intraperitoneal injection of butylate suppressed food intake in fasted mice and the effect was abolished with vagotomy [104].

### 7.2. Vagus Nerve-Mediated Pathways

Given the close physical proximity, gut bacteria can activate the vagus nerve, exerting effects on the brain. Specific bacterial strains regulate vagus nerve signalling to communicate with the brain, altering behaviour. Administration of a subclinical dose of the diarrhoea-causing pathogen *Campylobacter jejuni* increased anxiety-related behaviour and c-Fos immunoreactivity in vagal afferents and in the tractus solitarius nucleus [124]. Since expression of c-Fos gene is upregulated immediately after the stimulation of neurons [125], this result indicated the stimulation of vagal afferents. The beneficial effects of *Lactobacillus rhamnosus JB1* on anxiety- and depression-related behaviours were blocked via vagotomy [126]. *Lactobacillus reuteri* improved both the social behaviour in animal models of autism and wound healing in a vagus nerve-dependent manner [127]. The probiotic *B.longum* NC3001 reversed inflammation, colitis-induced anxiety and alteration of hippocampal *Bdnf* mRNA levels in mice [128]. The anxiolytic effects of *B.longum* NC3001 were absent in mice that had undergone vagotomy [128]. These findings demonstrated that the vagus nerve is one of the major signalling pathways from gut microbiota to the brain.

However, it is unclear whether the vagus nerve is activated by physical interaction with bacteria or by molecules produced by bacteria. While butyrate can activate vagal afferent nerve responses, the contribution of this interaction to the regulation of hippocampal BDNF expression is unknown [103,104]. The mechanisms by which vagal afferents are activated by the gut microbiota to increase hippocampal BDNF expression remain incompletely understood.

## 8. Peripheral Signalling and Hippocampal BDNF Levels in Aged Animals

Cognitive function gradually decreases with advancing age. Age-associated reduction in hippocampus volume is accompanied by altered dendritic branching, decreased dendritic spines and decreased adult neurogenesis [129]. BDNF has a central role in these processes, and its involvement in age-associated cognitive decline has been studied extensively [74,130,131,132,133]. Oh et al. interrogated microarray data from the orbitofrontal cortex of 209 healthy subjects, ranging from 16 to 96 years old, to identify the expression levels of both the *BDNF* gene and its specific receptor *TrkB*. The authors found that both expressions were downregulated in an age-associated manner [130]. Infusion of BDNF into the medial entorhinal cortex of aged rats for 28 days improved spatial memory [131]. Lentiviral gene delivery of BDNF into the entorhinal cortex restored age-related impairment of visuospatial leaning in aged rhesus monkeys [131]. Moreover, exercise improves both hippocampal BDNF expression and adult neurogenesis in aged animals [12,13]. Together, these reports indicate that depletion of BDNF/TrkB signalling contributes to the age-associated decline of cognitive function.

Since signalling through the vagus nerve affects constitutive expression levels of hippocampal *Bdnf* [36], it could be an important question whether vagal tone contribute to the age-associated regulation of hippocampal BDNF expression. We found that vagotomy had no effect on basal BDNF expression in the hippocampus of aged (15–16 months old) mice [18]. However, vagus nerve-mediated signalling elicited via CX3CL1 administration into the peritoneal cavity augmented BDNF expression in the hippocampus of aged mice [18]. Contrary to aged mice, young mice (6–7 weeks old) with vagotomy showed decreased basal BDNF expression in the hippocampus [36]. Considering the age-associated decrease in hippocampal BDNF expression [130], these findings potentially suggest that the effect of vagal tone on hippocampal BDNF expression decreases with ageing. Nevertheless, signalling through the vagus nerve still has the potential to upregulate the hippocampal BDNF expression in aged mice. This supports the notion that age-associated alterations in vagus tone contribute to age-associated decline in hippocampal BDNF expression.

Concerning the effects of the gut microbiota, hippocampal BDNF expression in aged rats (20–24 months old) was approximately 50% of that in young rats (3 months old) and faecal microbiota transplantation from aged rats into young rats decreased hippocampal BDNF expression levels to those in aged rats [74]. Coincidently, faecal transplantation from aged rats into young rats altered the gut microbiota composition of young rats, with pronounced increases in the bacteria populations abundant in aged rats, such as *Prevotella*, *Bacteroides* and *Parabacterioides* [74]. This suggests a close relationship between gut microbiota and hippocampal BDNF expression. In aged rats, administration of VSL #3, a probiotic mixture, suppressed inflammation by decreasing IL-10 expression and increased expression of BDNF and synapsin in the hippocampus [75]. A diet supplemented with *Lactobacillus paracasei* K71 improved cognitive performance in ageing-accelerated mice through the upregulation of hippocampal BDNF expression [76]. These reports demonstrate the possible involvement of the gut microbiota in the age-associated regulation of BDNF expression. However, these reports did not address the question of whether the vagus nerve is involved in the gut bacteria-induced alteration of hippocampal BDNF expression.

Taken together, signalling from peripheral tissues elicited through exercise, gut bacteria and intraperitoneal CX3CL1 administration are closely associated with the regulation of hippocampal BDNF expression in aged mice.

## 9. Conclusions

Accumulating evidence indicates the involvement of the gut microbiota in the regulation of BDNF expression in the hippocampus. Moreover, signalling elicited via the intraperitoneal administration of CX3CL1 promotes hippocampal BDNF expression in aged mice [18]. CX3CL1 induces phenotypic changes in the peritoneal cells of aged mice, and transplantation of the peritoneal cells prepared from aged mice treated with CX3CL1 was enough to improve novel object recognition memory impaired by advancing age. This finding potentially indicates that the gut microbiota is not the sole mechanism by which peripheral tissues regulate hippocampal BDNF expression. These reports indicate that signalling from peripheral tissues affects BDNF expression in the adult hippocampus.

The peripheral signalling via the vagus nerve is seemingly one of the mechanisms by which peripheral tissues regulate the constitutive level of hippocampal BDNF. The gut microbiota is a potential mechanism that corresponds to vagal tone regulation of constitutive hippocampal BDNF expression. However, most studies supporting this hypothesis have employed an inoculation of specific bacteria species. While some bacteria strains affect the regulation of hippocampal BDNF expression, the contribution of the vagus nerve has not been elucidated for most bacterial species and strains. Only a few specific bacteria strains are known to stimulate hippocampal BDNF expression in a vagus nerve-dependent manner [124,126,127,128]. The mechanism by which vagal tone is stimulated to upregulate constitutive level of hippocampal BDNF is still unknown.

Both peripheral tissue-initiated signalling across the blood–brain barrier and via the vagus nerve are implicated in age-associated changes of adult neurogenesis through the regulation of hippocampal BDNF expression. Moreover, beneficial effects of exercise are evident in aged animals. These findings might imply that signalling from peripheral tissues contributes to the ageing of brain function through the regulation of central BDNF expression. Further elucidating the relationship between vagus nerve-mediated hippocampal BDNF induction and blood factors regulating adult hippocampal neurogenesis is also essential to understand how peripheral tissues regulate hippocampal BDNF expression. Considering that the blood factors alter DNA methylation in NSCs in the hippocampus [14] and that Bdnf gene expression is regulated in part by the DNA methylation status [134], the effects of vagal nerve signalling on the DNA methylation status of cells in the hippocampus are an important future scientific question. Further studies are required for elucidation of the mechanism through which peripheral tissues regulate central BDNF expression.

## Figures and Tables

**Table 1:** Effects of gut bacteria on hippocampal BDNF expression.

Animal	Condition	Hippocampal BDNF Expression	Reference Number
NMRI mouse	Germ-free	↓	[65]
NIH Swiss mouse	Antibiotics	↓	[66]
Sprague Dawley rat	Bifidobacterium breve 6330	↑	[67]
Male ICR mouse	Lactobacillus johnsonii CJLJ103	↑	[68]
Male Wistar rat	Lactobacillus plantarum IS-10506	↑	[69]
Male Sprague Dawley rat	Prebiotics	↑	[70]
Female Swiss Webster mouse	Germ-free	↑	[72]
BALB/c mouse	Antibiotics	↑	[73]
NIH Swiss mouse	Germ-free and colonisation with gut bacteria of SPF ^#^ BALB/c mouse	↓	[73]
BALB/c mouse	Germ-free and colonisation with gut bacteria of SPF NIH Swiss mouse	↑	[73]
Young Sprague Dawley rat	Faecal microbiota transplantation from aged rat	↓	[74]
Aged Wister rat	A probiotics mixture VSL #3	↑	[75]
Senescence-accelerated mouse prone 8 (SAMP8)	Lactobacillus paracasei K71	↑	[76]

^#^ Specific pathogen free.

## Data Availability

Not applicable.
